# Transcranial Electrical Stimulation and Behavioral Change: The Intermediary Influence of the Brain

**DOI:** 10.3389/fnhum.2017.00112

**Published:** 2017-03-14

**Authors:** Siobhán Harty, Francesco Sella, Roi Cohen Kadosh

**Affiliations:** Department of Experimental Psychology, University of OxfordOxford, UK

**Keywords:** transcranial electrical stimulation, trancranial direct current stimulation, behavior, neurophysiology, mediation analysis, moderation analysis

## Introduction

Numerous studies have shown that transcranial electrical stimulation (tES) can modulate a wide-range of behavioral processes (Coffman et al., 2014; Harty et al., 2014; Sarkar et al., 2014; Pasqualotto, 2016), and ameliorate deficits in several neuropsychiatric disorders (for reviews see Kekic et al., 2016; Lefaucheur et al., 2017). These promising outcomes, in conjunction with the fact that the approach is safe and inexpensive, have generated enthusiasm for its viability as both an investigative and neuroenhancement tool. However, concerns about the variability and reproducibility of tES effects have constrained progression with its application (Jacobson et al., 2012; Berlim et al., 2013; Horvath et al., 2015). Many factors may contribute to the variability and poor reproducibility of findings. Some of these have already been discussed elsewhere such as insufficient statistical power, methodological differences across studies, experimenter error, inadequate sensitivity and test-retest reliability of the outcome measures (Horvath et al., 2015; Open Science Collaboration, 2015). However, one factor that we believe has received insufficient consideration to date concerns the extent to which the assumptions relating to the targeted brain region are supported (Bikson and Rahman, 2013; Miniussi et al., 2013; Plewnia et al., 2015; Harty et al., 2017). In the present article, we highlight the importance of accounting for states and traits of the neurophysiological milieu when assessing the effects of interventions such as tES on behavior. We present hypothetical scenarios relating to the use of transcranial direct current stimulation (tDCS), but the discussed logic equally applies to other electrical and magnetic stimulation techniques. We additionally propose that mediation and moderation analyses constitute valuable and elegant statistical approaches for assessing the dynamic interaction between these interventions, the brain, and behavior.

## A fundamental assumption of tDCS research

**Figure 1:**
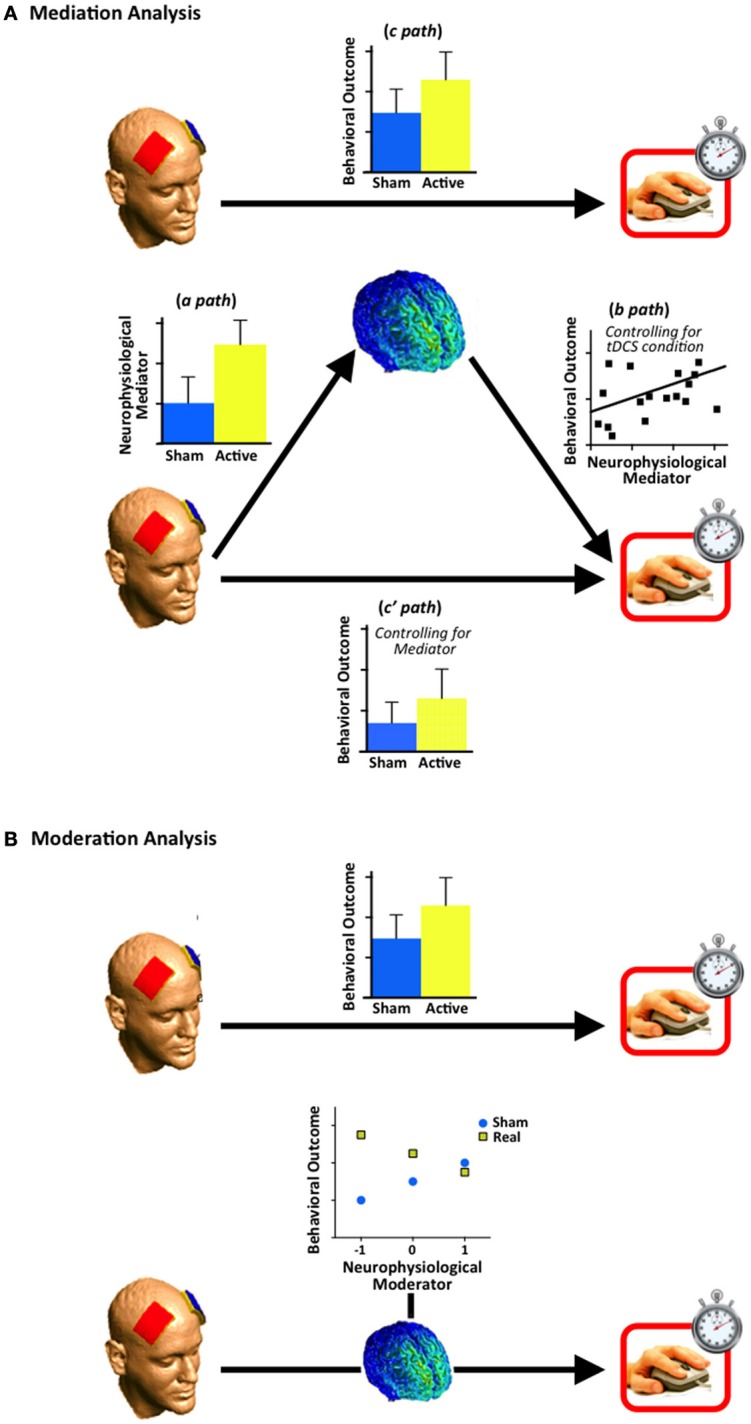
**Schematics for mediation and moderation analyses. (A)** Upper panel: A linear regression examining the relationship between transcranial direct current stimulation (tDCS; Active vs. Sham) and the behavioral outcome measure (*c path*). One can proceed to the analyses in the lower panel regardless of whether a significant relationship is observed here. Lower panel: The effect of tDCS condition on the neurophysiological index is evaluated with a linear regression (*a path*). The relationship between the neurophysiological index and the outcome variable is evaluated with another linear regression, which also includes the tDCS condition as a predictor (*b path*). The effect of tDCS condition on the outcome variable is re-evaluated using a linear regression that also includes the neurophysiological mediator as a predictor (*c' path*) The bar chart for the *c' path* represents the adjusted means when the impact of the neurophysiological mediator is controlled for. Finally, the mediation hypothesis is evaluated through one of the three approaches outlined in the main text; **(B)** Upper panel: A linear regression examining the relationship between tDCS condition and the behavioral outcome measure (*c path*). Lower panel: In accordance with standard convention for moderation analyses (Aiken and West, 1991), the estimated value of the outcome variable for each condition is reported at the mean, one standard deviation below the mean and one standard deviation above the mean, of the proposed moderating variable. This example shows a significant moderation effect: the impact of the tDCS condition on the behavioral outcome changes according to the value of the neurophysiological index (i.e., the moderator). Note that the heatmap shown for the neurophysiological index in both **(A,B)** is for illustrative purposes only, and does not reflect neural activity obtained from a neurophysiological assay.

The primary objective of most tDCS studies is to establish an association between the application of weak electric currents to specified locations on the scalp and changes in a behavioral index of interest. An implicit assumption of this approach is that the electric currents modulate neural activity in the regions beneath the scalp locations and accordingly affect behaviors supported by these neural regions. A corollary of this assumption has been that the efficacy of tDCS for modulating behavior has typically been evaluated by assessing the direct effect of tDCS (Active vs. Sham) on the behavior of interest (Figure 1A, top panel). A limitation of this approach is that it disregards the fact that the impact of tDCS on behavioral outcomes will inevitably depend on how the neurophysiological milieu of each individual responds to the tDCS. This is a particularly pertinent consideration given the growing literature demonstrating how various states and traits of the neurophysiological milieu can influence the impact of tDCS on behavior (Krause and Cohen Kadosh, 2014; Li et al., 2015). Accordingly, both tDCS *and the neurophysiological milieu* should be regarded as critical antecedents to tDCS-related behavioral effects. Given the pivotal role of the neurophysiological milieu in determining tDCS-related behavior outcomes, we propose that relevant neurophysiological measures should be acquired and accounted for more routinely when examining the efficacy of tDCS for modulating a given behavior. We furthermore advance that the mediating and moderating roles of the neurophysiological milieu can be efficiently evaluated using mediation and moderation analyses (Hayes, 2009).

## Conceptual overview of mediation and moderation analyses

### Mediation analysis

Mediation analysis is a form of regression that can be used to simultaneously evaluate the direct effect of tDCS on behavior and the indirect effect of stimulation on behavior through the brain. In the simplest version of this statistical model, the tDCS condition (e.g., Active vs. Sham) is the independent variable, an implicated brain index constitutes the mediating variable, and the behavioral index of interest constitutes the outcome variable (Figure 1A).

First, we determine whether there is a significant difference in the behavioral index for each tDCS condition (called *c path)*, as indexed by simple linear regression. This relationship represents the direct effect of stimulation on behavior, and the majority of tDCS studies to date have focused solely on this bivariate relationship. Second, we investigate whether there is a significant difference in the brain index for each tDCS condition (called *a path)*. A significant relationship here implies that the implicated brain index was significantly modulated by tDCS. Next, we evaluate whether the brain index (mediating variable) is a significant predictor of the behavioral index (*b path*) when tDCS condition is also included in the model (called *c' path*).

Finally, the mediation hypothesis is evaluated. The three most common approaches for determining whether there is a mediation effect are the following: (1) establish that the regression coefficients for the *a path* and the *b path* are both significant different from zero (test of joint significance; Kenny et al., 1998); (2) use bootstrapping with replacement to derive a distribution of the product of the *a path* and *b path* regression coefficients, and confirm that the 95% confidence intervals of the distribution do not overlap zero (Hayes, 2009; Mackinnon and Fairchild, 2009); or (3) determine that the product of the regression coefficients from the *a path* and *b path* is significantly different from zero when evaluated using the Sobel test (for details, see Sobel, 1986). If a mediation effect is established, it can be claimed that the proposed mediating brain index mediates the relationship between tDCS condition and behavior.

To underscore the value of measuring theoretically implicated neural indices and including them in mediation analyses, we provide the following simplified hypothetical research scenario. Let us assume that we are interested in determining the effect of tDCS applied to the dorsolateral prefrontal cortex (dlPFC) on working memory, which is assumed to rely on the dlPFC (Brunoni and Vanderhasselt, 2014). The prevailing approach to determine an effect in this context would be to examine the effect of tDCS (Active vs. Sham) on working memory performance using some form of a bivariate analysis. This analysis may or may not reveal a tDCS-related change in working memory performance. The lack of a behavioral change will likely leave us pondering several different possibilities about why no effect was observed. For example, we might wonder whether or not we succeeded to stimulate the targeted brain region. And, if not, was this attributable to one of the many parameters of the tDCS protocol (e.g., intensity or duration of the stimulation, size or location or the electrodes) being unsuitable? We might also have doubts regarding our assumption about the involvement of the targeted region to begin with. We might question whether our potential to pick up on a main effect was hampered by variability in the flow and distribution of the electric current across subjects, or by one of the many other inter-individual differences that are known to affect responsiveness to tDCS (e.g., Krause and Cohen Kadosh, 2014; Li et al., 2015). Similarly, we might contemplate whether different individuals within the study sample could have employed different cognitive strategies, and in turn different brain regions, to carry out the task. Variation in strategy use is a particularly pertinent consideration when appraising the effects of tDCS on behavior as we know that the currents involved in tDCS will not elicit neural firing. Rather, they only modulate the likelihood of firing within populations of neurons that are already naturally engaged by ongoing activity. Therefore, any tDCS-related effects on behavior are critically contingent on subjects' intrinsic recruitment of the target brain region to perform the task. We are thus left with several different questions that cannot be resolved when behavioral indices are our only outcome measure.

In contrast, by quantifying the response of the dlPFC to tDCS with an appropriate neurophysiological assay (e.g., pre- to post- change in blood-oxygen level-dependent (BOLD) response), and including this in a mediation analysis we can gain insights to inform many of these questions. For instance, the assumption about the role of the dlPFC in working memory, the assumption that the employed tDCS protocol is successfully modulating this area, and the extent to which this is common across subjects can all be verified. It is important to underscore that an initial significant direct effect (*c path*) is not a critical requisite for advancing with a mediation analysis (Hayes, 2009). For instance, we may not observe a direct effect of tDCS on working memory, but by pursuing with the mediation analysis we may find that tDCS was associated with an increase in activity in the dlPFC (*a path*) and this change in activity was in turn associated with an improvement in working memory (*b path*). If we substantiate the mediation hypothesis through one of the aforementioned approaches, we can formally infer that the tDCS-related change in dlPFC activity mediated the tDCS-related change in working memory. This hypothetical example serves to demonstrate how it is imperative to be cautious about drawing conclusions about the efficacy of tDCS for modulating behavior with the prevailing bivariate analyses. This point is particularly relevant when only small to medium sample sizes are under question, which has been the case for the vast majority of tDCS studies to date. Furthermore, this example highlights how the systematic assessment of theoretically implicated brain indices and their inclusion in a mediation model could reduce the chances of spurious conclusions in tDCS research.

### Moderation analysis

Moderation analysis is also a form of regression analysis, but here the objective is to determine whether the relationship between the independent and dependent variables changes as a function of a third variable (i.e., statistical interaction), known as the moderator (Figure 1B). Thus, while mediation analyses can provide insight on how behavioral effects are achieved (e.g., a change in activity within the neurophysiological milieu), moderation analyses can determine particular conditions for which the effects will hold. In the context of the hypothetical experiment described above, it is plausible that an effect of tDCS, or lack thereof, on working memory performance may be driven by a subset of subjects who had particular baseline neurophysiological characteristics, such as, for example, lower than average gray matter (GMD) density in the dlPFC. Here, moderation analyses could provide an elegant unified framework for demonstrating that the relationship between tES and behavior is moderated by individual differences in the GMD of the targeted region. Accordingly, we would be able to make a more refined interpretation regarding the efficacy of tDCS: the reported effect of tDCS applied over dlPFC on working memory was particular to a select group of individuals with low GMD in the target region. Identifying these kinds of caveats has important implications for the translational potential of tDCS research and the development of individualized protocols.

In summary, most tDCS research is based on the assumption that weak direct currents applied to the scalp will stimulate the underlying brain regions, resulting in a detectable change in associated behavioral indices. However, we have argued that this and other assumptions need to be formally verified by acquiring data regarding the actual states and traits of the targeted neural region. We suggest that the inclusion of theoretically implicated neurophysiological indices in mediation and moderation models constitute valuable approaches for enhancing the inferential power of tDCS research, by revealing how and for whom tDCS is effective. Exploiting these approaches should also yield information for guiding the design of more effective and personalized tDCS protocols. More generally, the nuanced insights that these approaches afford should reduce the likelihood of spurious conclusions, and accordingly improve the prospects for reproducibility in the field.

On a final note, mediation and moderation analysis can be readily implemented using open source plug-ins for common statistical software packages such as SAS, SPSS (e.g., Process by Hayes, 2013, MEMORE by Montoya and Hayes, 2017), and R (e.g., The Lavaan package; Rosseel, 2012). In addition to the basic forms of the mediation and moderation models we discussed here, these plug-ins provide other analysis templates with varying levels of complexity, including moderated mediation and mediated moderation, as well as options to incorporate multiple mediators, moderators and covariates.

## Author contributions

SH wrote the article and helped to conceive the opinion. FS helped to conceive the opinion and provided feedback on drafts of the article. RK helped to conceive the opinion and provided feedback on drafts of the article.

## Funding

This work was supported by grants from the James S. McDonnell Foundation 21st Century Science Initiative in Understanding Human Cognition and the European Research Council (Learning and Achievement; 338065).

### Conflict of interest statement

The authors declare that the research was conducted in the absence of any commercial or financial relationships that could be construed as a potential conflict of interest.
